# Supplementary figures and images for: Regulation of innate immune responses in macrophages differentiated in the presence of vitamin D and infected with dengue virus 2
Source: PLoS Negl Trop Dis. 2021 Oct 11;15(10):e0009873. doi: 10.1371/journal.pntd.0009873 (PMC8530315; doi:10.1371/journal.pntd.0009873)

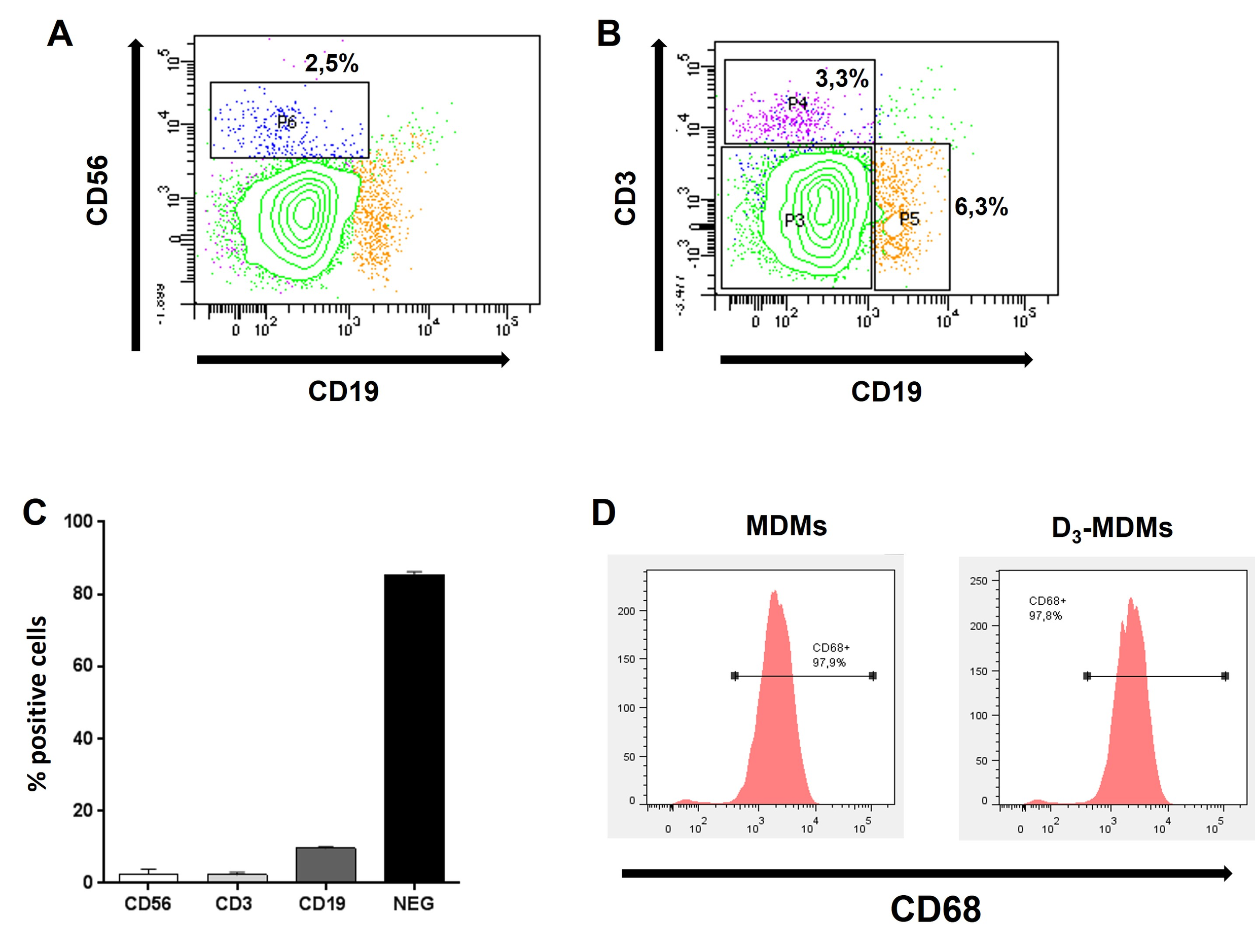

Supplement: S1 Fig — Monocytes were purified from PBMCs, and purity was assessed by the quantification of contaminant CD19+, CD3+ and CD56+ (A, B and C). In D, a representative histogram with CD68 expression levels of monocyte-derived macrophages (MDMs) and D3-MDMs after 6 days of differentiation is shown. Figure in C represent 3 individual experiments. (TIF) [file pntd.0009873.s001.tif]

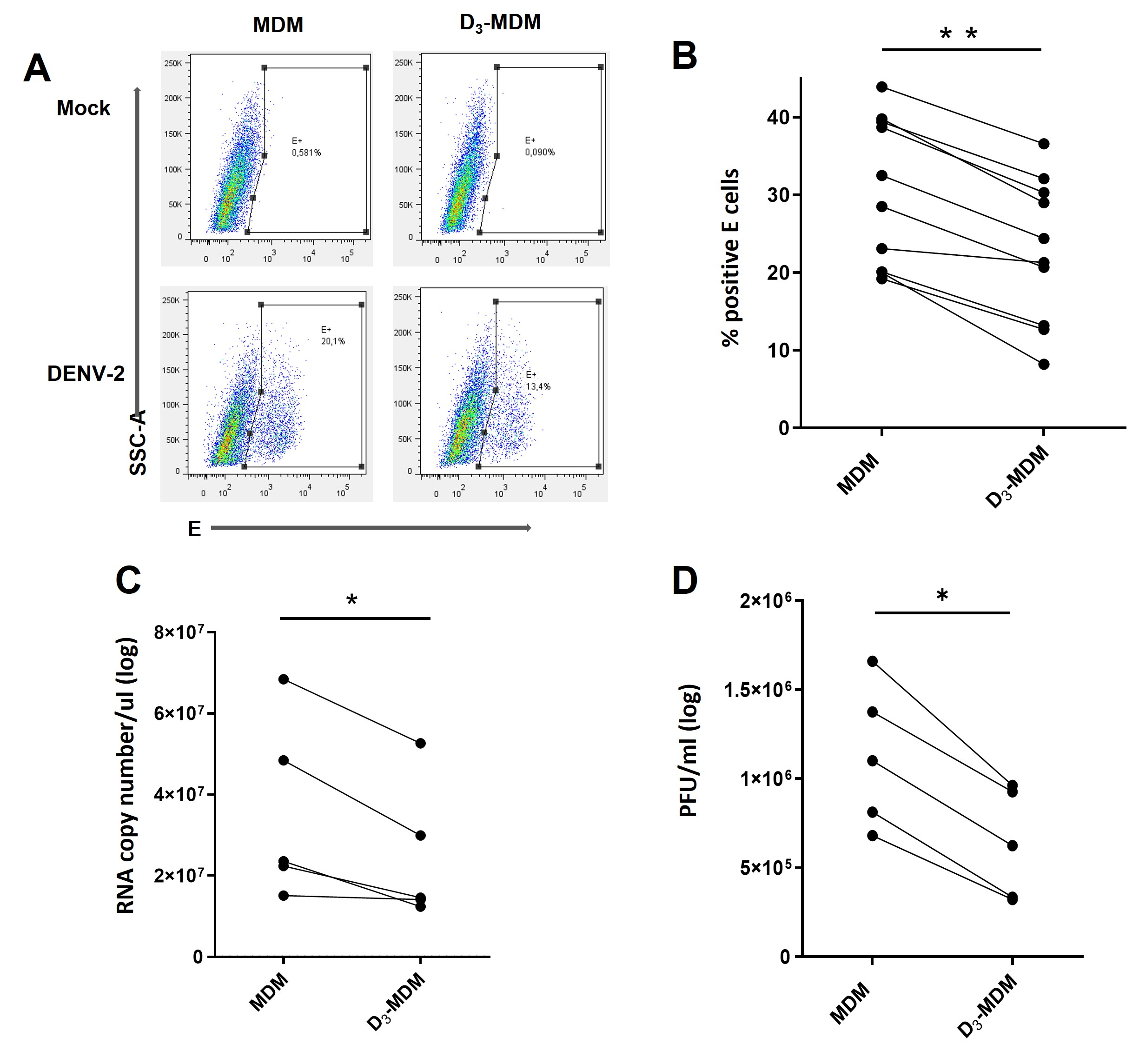

Supplement: S2 Fig — MDMs were differentiated in the presence of VitD3 (0.1 nM) for 6 days (D3-MDMs) and infected with DENV-2 with an MOI of 5 for 24 h. Infection was evaluated through flow cytometry and expressed as % E-positive cells. In A, a representative dot-plot of an individual experiment is shown, while B shows a summary of the results. Replication of DENV-2 was evaluated by quantification of intracellular viral RNA copy number using qPCR (C) and by quantification of viral titer in the supernatant using a plaque assay (D). Figures represent 10 individual experiments from different donors (B), while five individual experiments are shown in C. Differences were obtained with a Wilcoxon test using a 95% confidence interval (**p < 0.01, *p < 0.05). (TIF) [file pntd.0009873.s002.tif]

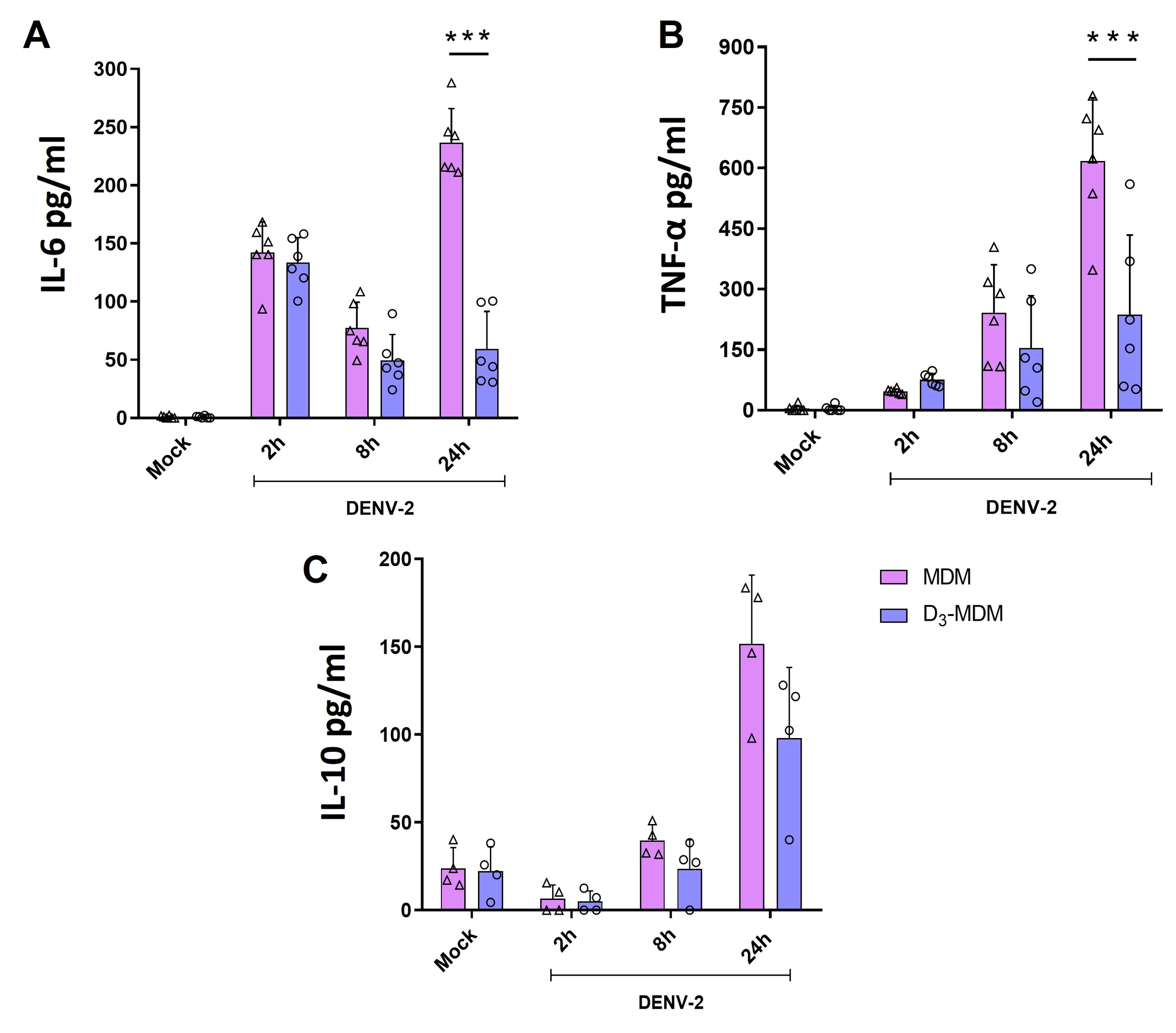

Supplement: S3 Fig — MDMs were differentiated in the presence of VitD3 (0.1 nM) for 6 days (D3-MDMs) and then infected with DENV-2 with a MOI of 5 for 2, 8, or 24h. Production of IL-6 (A), TNF-α (B), and IL-10 (C) was measured by ELISA in MDMs and D3-MDMs. Figures represent six individual experiments from different donors (A and B), while four individual experiments are shown in C. Differences were obtained using a two-way ANOVA with a Bonferroni post-hoc test using a 95% confidence interval (*** p < 0 .001) (TIF) [file pntd.0009873.s003.tif]

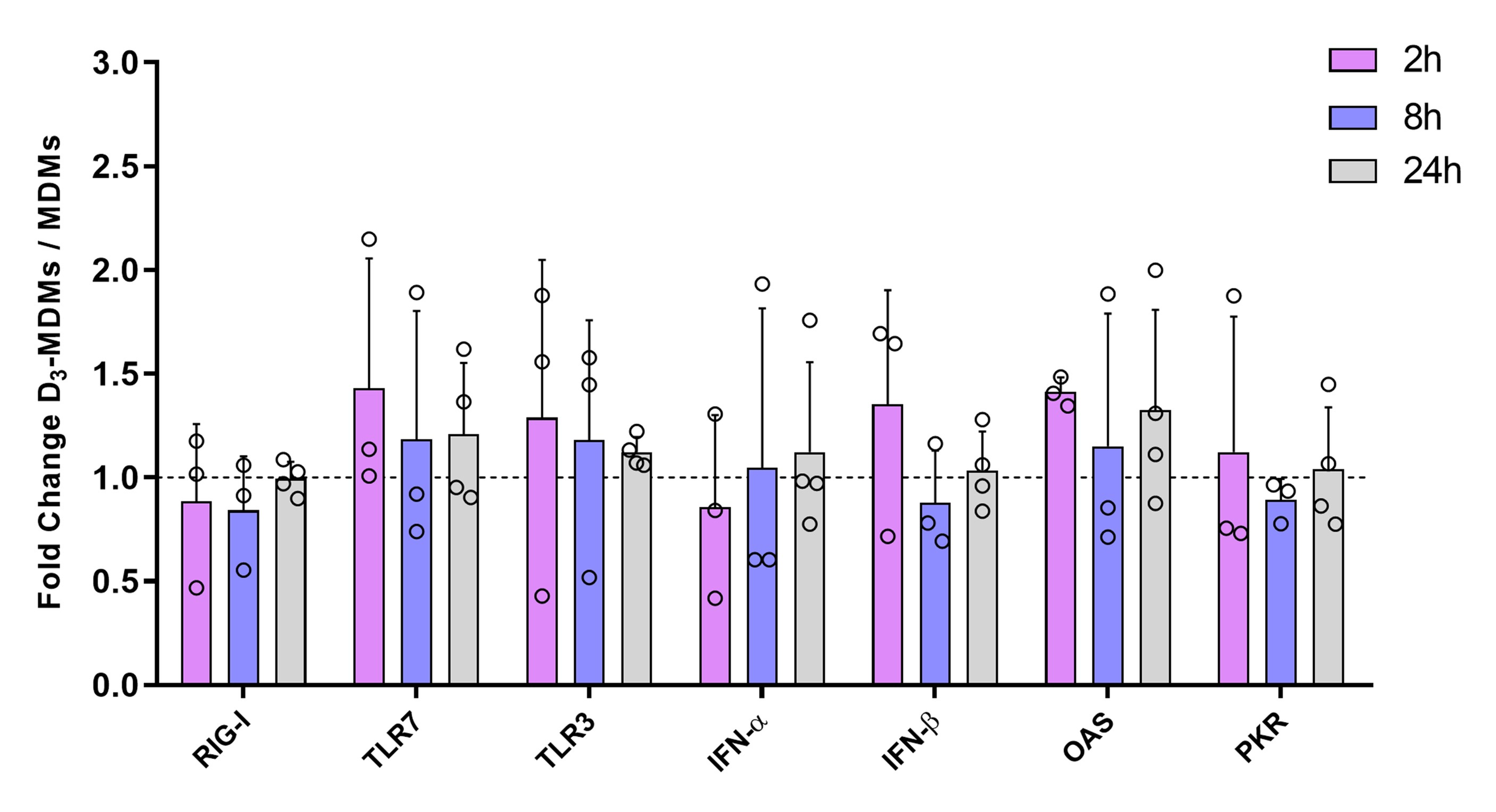

Supplement: S4 Fig — MDMs were differentiated in the presence of VitD3 (0.1 nM) for 6 days (D3-MDMs) and then expression of RIG-I, TLR7, TLR3, IFN-α, IFN-β, PKR and OAS1 were measured by qPCR in MDMs and D3-MDMs using ubiquitin-conjugating enzyme E2D2 as housekeeping. Data is expressed as fold change relative to mock treated MDMs. Figures represent three individual experiments from different donors. Differences were obtained with a two-way ANOVA with a Bonferroni post-hoc test and a 95% confidence interval (***p < 0.001, **p < 0.01, *p < 0.05). (TIF) [file pntd.0009873.s004.tif]

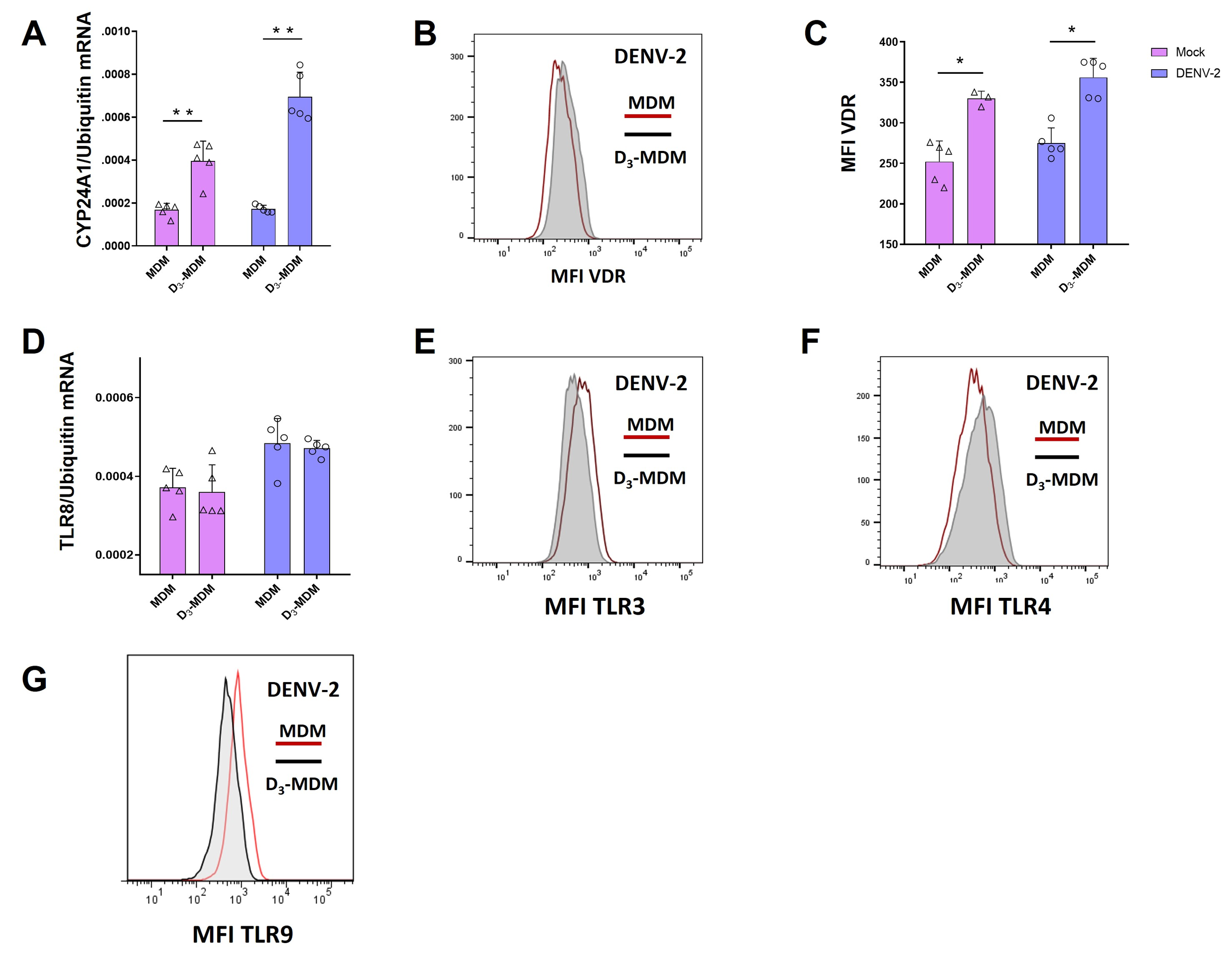

Supplement: S5 Fig — MDMs were differentiated in the presence of VitD3 (0.1 nM) for 6 days (D3-MDMs) and then infected with DENV-2 with a MOI of 5 for 24 h. Expression of CYP24A1 (A) and TLR8 (D) were measured by qPCR in MDMs and D3-MDMs both infected and mock-infected with DENV-2, using the gene encoding ubiquitin-conjugating enzyme E2D2 as a housekeeper gene. Data is expressed as relative expression normalized to the expression level of the housekeeper gene. Expression at the protein level of VDR (B), TLR3 (E) TLR4 (F) and TLR9 (G) was measured by flow cytometry and expressed as mean fluorescence intensity. Figures represent five individual experiments from different donors. Differences were obtained with a two-way ANOVA with a Bonferroni post-hoc test and a 95% confidence interval (**p < 0.01, *p < 0.05). (TIF) [file pntd.0009873.s005.tif]
