# Supplementary material for: Regulation of innate immune responses in macrophages differentiated in the presence of vitamin D and infected with dengue virus 2
Source: PLoS Negl Trop Dis. 2021 Oct 11;15(10):e0009873. doi: 10.1371/journal.pntd.0009873 (PMC8530315; doi:10.1371/journal.pntd.0009873)
Supplement: S1 Table — (DOCX) [file pntd.0009873.s006.docx]

**Supplemental Table 1.**

| Gene | | Sequence 3´---5´ |  |
| --- | --- | --- | --- |
| *TLR3* | | Fw: GTCAGATTTAAACATTCCTCTTCGC |  |
|  |  | Rv: ATTGGGTCTGGGAACATTTCTCTTC |  |
| *TLR4* | | Fw: GCCCTGCGTGGAGGTGGTTCCTA |  |
|  |  | Rv: AGCTGCCTAAATGCCTCAGGGGAT |  |
| *TLR7* | | Fw: TTACCTGGATGGAAACCAGCTACT |  |
|  |  | Rv: TCAAGGCTGAGAAGCTGTAAGCTA |  |
| *TLR9* | | Fw: TTATGGACTTCCTGCTGGAGGTGC |  |
|  |  | Rv: CTGCGTTTTGTCGAAGACCA |  |
| *DDX58* (RIG-I) | | Fw: AGGAAAACTGGCCCAAAACT |  |
|  |  | Rv: TTTCCCCTTTTGTCCTTGTG |  |
| *IFNA1* (IFN-α) | | Fw: CAGAGTCACCCATCTCAGCA |  |
|  |  | Rv: CACCACCAGGACCATCAGTA |  |
| *IFNB1* (IFN-β) | | Fw: CGCCGCATTGACCATCTA |  |
|  |  | Rv: GACATTAGCCAGGAGGTTTCTCA |  |
| *EIF2AK2* (PKR) | | Fw: GGTACAGGTTCTACTAAACA |  |
|  |  | Rv: GAAAACTTGGCCAAATCCACC |  |
| *OAS1* | | Fw: GTGTGTCCAAGGTGGTAAAGG |  |
|  |  | Rv: CTGCTCAAACTTCACGGAA |  |
| *VDR* | | Fw: TGCTATGCACTGTGAAGGCGT |  |
|  |  | Rv: AGTGGCGTCGGTTGTCCTT |  |
| *CYP24A1* | | Fw: CGCAAATACGACATCCAGGC |  |
|  |  | Rv: AATACCACCATCTGAGGCGT |  |
| *SOCS1* | | Fw: CACTTCCGCACATTCCGTTC |  |
|  |  | Rv: CACGCTAAGGGCGAAAAAGC |  |
| DENV-2 | Fw: CAATATGCTGAAACGCGAGAGAAA | | |
|  | Rv: CCCCATCTATTCAGAATCCCTGCT | | |
| *UBE2D2* (ubiquitin-conjugating enzyme E2D2 | Fw: CCCTTCAAACCACCTAAGGTTGC | | |
|  | Rv: GTGCTGGAGACCACTGTGATCG | | |
