# Supplementary material for: Regulation of innate immune responses in macrophages differentiated in the presence of vitamin D and infected with dengue virus 2
Source: PLoS Negl Trop Dis. 2021 Oct 11;15(10):e0009873. doi: 10.1371/journal.pntd.0009873 (PMC8530315; doi:10.1371/journal.pntd.0009873)
Supplement: Attachment — Submitted filename: Response to reviewers.docx [file pntd.0009873.s007.docx]

**Answer to Reviewers**

**Reviewer #1:** In the present work, Castillo JA., et al. investigated the effect of Vit-D3 on the innate immune responses against DENV-2. They found that replication of DENV-2 in VitD3 treated monocyte-derived macrophages was restricted. They showed that VitD3 treatment downregulated the expression levels of TLRs, RIG but increased levels of SOCS-1, reduced ROS production related to the lower expression of TLR-9. Surprisingly, VitD3 did not modulate expression of IFN-α and IFB-β but higher expression of PKR and OAS1 mRNA were found in VitD3-MDM. Comments

1. Fig. 1 authors monitored the expression levels of RIG-I, TLR-3 and TLR-7 mRNA and the levels of TLR3, TLR7 protein productions. As shown in Fig.1C, mRNA of TLR-3 were peaked at 8 hr of infection in MDM treated and untreated with VitD3 while there was no change of TLR3 at 2 h of infection. Surprisingly, TLR-3 protein production peaked at 2 hr of infection (Fig. 1D). How can protein production reach the highest level earlier than its mRNA expression? This point need to be clarified.

***Answer:***

*We thank the reviewer for a draw into attention this particular result. Indeed, we found discrepancies between the kinetics of mRNA and protein expression of TLR3, and unfortunately, potential explanatory mechanisms remain speculative. However, some explanations were included in the discussion section (lines 432 to 445) and include analysis from transcriptome and proteomic studies* [1,2]*; changes in tRNA abundance due to stress conditions* [3]*; or the existence of pre-formed TLR3 complexes that could be recruited to endosomes rapidly after DENV recognition, similar to TLR2 recruitment to lipid rafts after recognition with other types of ligands* [4]*.*

2. Fig.2 means of % suppression at each MOI should be added in the Result section. In addition, differences of those means should be statistically compared among MOI.

***Answer:***

*To answer the reviewer, we have made such analyses and were included in the results section and Figure 2B (lines 329 to 334). Statistical differences in such % of suppression of infection induced by Vitamin D with each MOI were calculated.*

3. On page 8, 2nd paragraph, Fig. 3A-B, authors conclude that VitD3 regulated expression of TLRs independently of the MOI used. ……………..Taken together, our results suggest that…………………….in response to DENV-2 infection, possibly depending on the time of infection (early for TLR3, and later for TLR7). This point should be clarified. The author concluded that the response does not dependent on concentration of infection dose but depends on time of infection. The explanation should be added such as potential mechanism.

***Answer:***

*Following the Reviewer's comments, we clarified this point and a better explanation was added (lines 340 to 342). We want to point out that VitD3 regulates the expression of TLR3 and TLR7 in matched-infected samples demonstrating that regulation of these TLRs by VitD3 is a direct effect of the treatment and not of an effect consequence of a lower viral replication. Further, the analysis suggested by the reviewer in the previous item, included in the text, already helped to give more clarity to the text.*

4. In Discussion, a potential diagram showing how VitD3 suppress innate responses should be included.

***Answer:***

*Following the reviewer comments, a hypothetical model of the action of VitD3 in D3-MDMs was added (Fig. 7).*

**Reviewer #2:** This study examines the immunomodulatory effects of bioactive vitamin D3 metabolite (Vit.D3) in DENV-2-infected human macrophages. The authors show that monocytes differentiated into macrophages (MDMs) in the presence of VitD3 exhibit decreased expression of pattern recognition receptors (e.g. TLR3, TLR7, RIG-I) and elevated transcriptional activity of some IFN-stimulated genes, upon DENV-2 challenge. Overall, this is a strong study that reinforces the immunomodulatory role of vitamin D.  However, there are some aspects of it that appear to show a lack of rigor including the statistical analysis. Once these are dealt with appropriately the manuscript would be far more convincing.

Methods section:

a) Statistical analyses are poorly described and need to be revised. There is no mention of the Mann-Whitney test used in Fig. S1 and Fig. 2 and the normality assessment that justify the employment of such unpaired non-parametric test. In Fig. S1, the use of the Mann-Whitney test for matched-pair comparisons is inappropriate. Also, authors should check the use of two-way ANOVA to compare mean differences between MDMs vs. D3-MDMs at the same time point.
b) Flow cytometry was used to assess the expression pattern of VDR and DHR beyond TLR3/4/9 and DENV E antigen. This needs to be mentioned.
c) In table S1, use the gene nomenclature to name PCR targets for which primers were designed.

***Answer:***

*We thank the reviewer for this constructive comment. We are sorry for the confusion. The appropriate statistical test for Figure S1 was changed to Wilcoxon (matched-paired samples). Also, the statistical test for Figure 2 was changed for the appropriate two-way ANOVA, as it was already done for the rest of the Figures in the manuscript. The methods section (lines 273 to 275) was changed for including a description of VDR and DHR quantification in the flow cytometry section of M&M (line 217 to 220). Finally, the name of genes tested by qPCR and shown in Table S1 has changed and used accordingly to the gene nomenclature.*

Results:

a) The authors have done a ton of fine work, but in my opinion, they should improve the way the results are presented in order to make them easier to understand. My suggestions are: (i) standardize supplementary and main figures, plotting mean and SEM (or SD) plus individual values; (ii) label title to figures and then make visual separation of qPCR and flow cytometry results clearly. Labeling Y-axis differently [e.g. PKR/Ubiquitin mRNA (Fold-change of mock controls)] may be useful; (iii) present dot- and/or MFI-plot along with flow cytometry results; (iv) consider including mock-infected controls in each graph as done in panel 1D; (v) clarify in figure legends what each statistical hypothesis was being tested for; and (vi) indicate statistically and non-statistically significant differences in each figure panel (including S1D).

***Answer:***

b) *We thank the reviewer for his suggestions. All main and supplementary figures have been changed following the reviewer´s suggestions: (i) we plotted mean, SD, and individual values; (ii) we included title to each figure for making it easier to read; (iii) MFI-plots of flow cytometry data are presented in supplementary figure 5. (iv) data of mock-infected samples were included in the Supp. Figs. (v) In the legend of figures were included the statistical tests. (vi) the statistically significant differences were included in each figure; however, we have not included non-statistically significant because we consider that the figures become too loaded with information.*
c) Empirical data demonstrating the purity of MDMs is missing. Authors should include it as supplementary material.

***Answer:***

*Data showing purity of monocytes before differentiation, and purity of MDMs after differentiation is now described in the Methods section (line 167 to 170), results section (line 285 to 288) and shown in supplementary figures 1A to 1D.*

d) Why did the authors use only MFI to infer protein levels? Is there any difference concerning the number of positive cells for a given target between MDMS and D3-MDMs?

***Answer:***

*We thank the reviewer for his question. We analyzed flow cytometry data in terms of % of positive cells and MFI to determine the protein level of TLRs evaluated. However, the number of cells that express the TLRs evaluated is large thus making it difficult to analyze differences. For example, in the case of TLR3, at 2 hpi., 98,9% and 92,9% of MDMs and D3-MDMs, respectively, were positive. MDMs express a wide array of PRRs for recognizing and mediating innate immune responses against invading pathogens. Therefore, it is expected that the majority of cells express the TLRs we evaluated. Nevertheless, changes in the MFI of these TLRs could have an effect on the quantity and quality of activation of MDMs and in the subsequent inflammatory response.*

e) 3rd paragraph “ …observed between these types of cells”: they are all macrophages.

***Answer:***

*The sentence was modified (lines 321 to 322).*

f) Fig 1A and 1D: How do the authors explain the early reduction of TLR3 after 2hpi with DENV-2 since mRNA level is changed by 24hpi?

***Answer:***

*The same question was answered to reviewer # 1 (lines 432 to 445)*

g) Mention is made of ELISA assays for IL-6, IL-10, and TNF but no data are presented in the manuscript. I would really like to see this data. It would confirm the results previously published by the group (Ref. 17) and reinforce the main claims of this study.

***Answer:***

*We appreciate the reviewer for this request, and we are sorry for this. Data regarding cytokine production by MDMs and D_3_-MDMs during DENV infection was added in the results section (lines 292 to 295) and shown in supplementary figure 3.*

DISCUSSION

a) Could the data collected for DENV-2 be extended for other DENV serotypes? Authors should comment on this.

***Answer:***

*This suggestion given by the reviewer is very interesting. A paragraph addressing this question has been included in the discussion section (line 535 to 542).*

b) There is no evidence that the authors see any limitation to their own work. They should add a paragraph on limitations of the work presented.

***Answer:***

*We appreciate the reviewer for this suggestion. Accordingly, we have included some limitations of the study throughout the discussion section. Specifically, we see a limitation in the lack of data about TLR7 expression at the protein level, and the lack of data regarding TLR2 expression. Similarly, we addressed the limitation of data only with DENV-2, but this particular issue was resolved in the previous item.*

***References***

1.	Kawaguchi R, Bailey-Serres J. Regulation of translational initiation in plants. Curr Opin Plant Biol. 2002;5: 460–465. doi:10.1016/S1369-5266(02)00290-X

2.	Gygi SP, Rochon Y, Franza BR, Aebersold R. Correlation between protein and mRNA abundance in yeast. Mol Cell Biol. 1999;19: 1720–1730.

3.	Torrent M, Chalancon G, De Groot NS, Wuster A, Madan Babu M. Cells alter their tRNA abundance to selectively regulate protein synthesis during stress conditions. Sci Signal. 2018;11: 1–10. doi:10.1126/scisignal.aat6409

4.	Triantafilou M, Gamper FGJ, Haston RM, Mouratis MA, Morath S, Hartung T, et al. Membrane sorting of toll-like receptor (TLR)-2/6 and TLR2/1 heterodimers at the cell surface determines heterotypic associations with CD36 and intracellular targeting. J Biol Chem. 2006;281: 31002–31011. doi:10.1074/jbc.M602794200
